# Social learning promotes nicotine self-administration by facilitating the extinction of conditioned aversion in isogenic strains of rats

**DOI:** 10.1038/s41598-017-08291-5

**Published:** 2017-08-14

**Authors:** Wenyan Han, Tengfei Wang, Hao Chen

**Affiliations:** Department of Pharmacology, University of Tennessee Health Science Center, 71 S. Manassas St., Room 205 Translational Science Research Building, Memphis, TN 38163 USA

## Abstract

Both social environment and genetic factors are critical for smoking initiation and nicotine addiction. We reported that rats developed conditioned flavor (i.e., taste and odor) aversion to intravenously self-administered (IVSA) nicotine, and that social learning promoted nicotine IVSA with flavor cues. We thus tested the hypothesis that socially acquired nicotine IVSA is a heritable trait by using female rats of six inbred strains and six F1 hybrids. Each strain was tested for 10 daily IVSA sessions. We found that the intake of nicotine (15 and 30 μg/kg/inf) varied among these strains by 33.7–56.6-fold. The heritability of nicotine intake was estimated to be 0.54–0.65. Further, there was a strong correlation in nicotine intake (R^2^ = 0.85, p < 0.0001) between the two nicotine doses. Another cohort of rats was given three daily IVSA sessions followed by five sessions that tested conditioned flavor aversion. Nicotine intake was highly correlated with the extinction of the conditioned aversion (R^2^ = 0.58, p < 0.005). These data showed that nicotine intake in the socially acquired nicotine self-administration model is controlled by genetic factors and that the role of social learning is likely in facilitating the extinction of conditioned aversive response to nicotine.

## Introduction

Nicotine is the principal psychoactive ingredient of tobacco products^1^. It exerts marked reinforcing effects and maintains drug-seeking behavior^2, 3^. Paradoxically, nicotine is also aversive within the dose range where it is reinforcing. For example, doses of nicotine that maintained a high rate of responding in squirrel monkeys also induced vomiting^4^. In humans, the initial smoking experience is usually unpleasant and accompanied by physiological symptoms such as coughing, dizziness, and nausea^5^. The aversive nature of nicotine indicates that smoking initiation is likely influenced by other factors that could reduce the negative motivational effect of nicotine. One example of such a factor is the social environment. Not only is peer smoking the most significant predictor of smoking initiation^6^, but social interaction also increased the perceived reward of smoking^7^.

We previously established a model of socially acquired nicotine intravenous self-administration (IVSA) with an olfactogustatory cue in rats. We found that nicotine IVSA with a contingent appetitive oral flavor cue (i.e., taste and odor) resulted in conditioned flavor aversion (CFA)^8^. We also reported that social learning in this model was mediated by two factors: the odor (but not the taste) component of the flavor cue^8^ and carbon disulfide^9^, a component of mammalian breath.

Genetic factors contribute to approximately half of the variation in smoking behavior^10, 11^. Many studies have confirmed that variations in the CHRNA3-CHRNA5-CHRNB4 gene cluster are associated with smoking, particularly the number of cigarettes consumed per day^12^. Genes in this cluster, especially CHRNA5, have been shown to underlie the aversive effect of nicotine^13, 14^. However, this cluster accounted for only a small fraction of the heritability for smoking. Because of the large effect of social environment on smoking behavior, we hypothesized that genetic factors also play a role in this socially acquired nicotine IVSA model.

Although mouse has been the species of choice for behavioral genetic studies, the rat is emerging as a viable alternative, especially when complex behavioral paradigms are used^15^. We previously reported that genetic factors contributed to nicotine IVSA and food reward by using a lever press model in isogenic strains of adolescent rats^16^. Using female rats of the same panel of isogenic strains, we tested the above hypothesis using the socially- acquired nicotine self-administration model. Our results showed that nicotine intake in this model was highly heritable. In addition, we found a significant correlation between nicotine intake and the extinction of nicotine-conditioned aversion.

## Results

### Socially-acquired nicotine IVSA among 12 isogenic strains

IVSA, starting between postnatal day 41 and 44, were conducted in operant chambers that were divided by a partition. The partition had a row of six holes that allowed orofacial interaction between the two rats placed on each side. A flavor cue was provided to the self-administration rat via one of the two spouts (i.e., active) upon completing a fixed ratio 10 reinforcement schedule. Intravenous nicotine was delivered at that same time as the flavor cue. The flavor cue was also available without restriction on the side where a demonstrator rat was placed.

**Figure 1:**
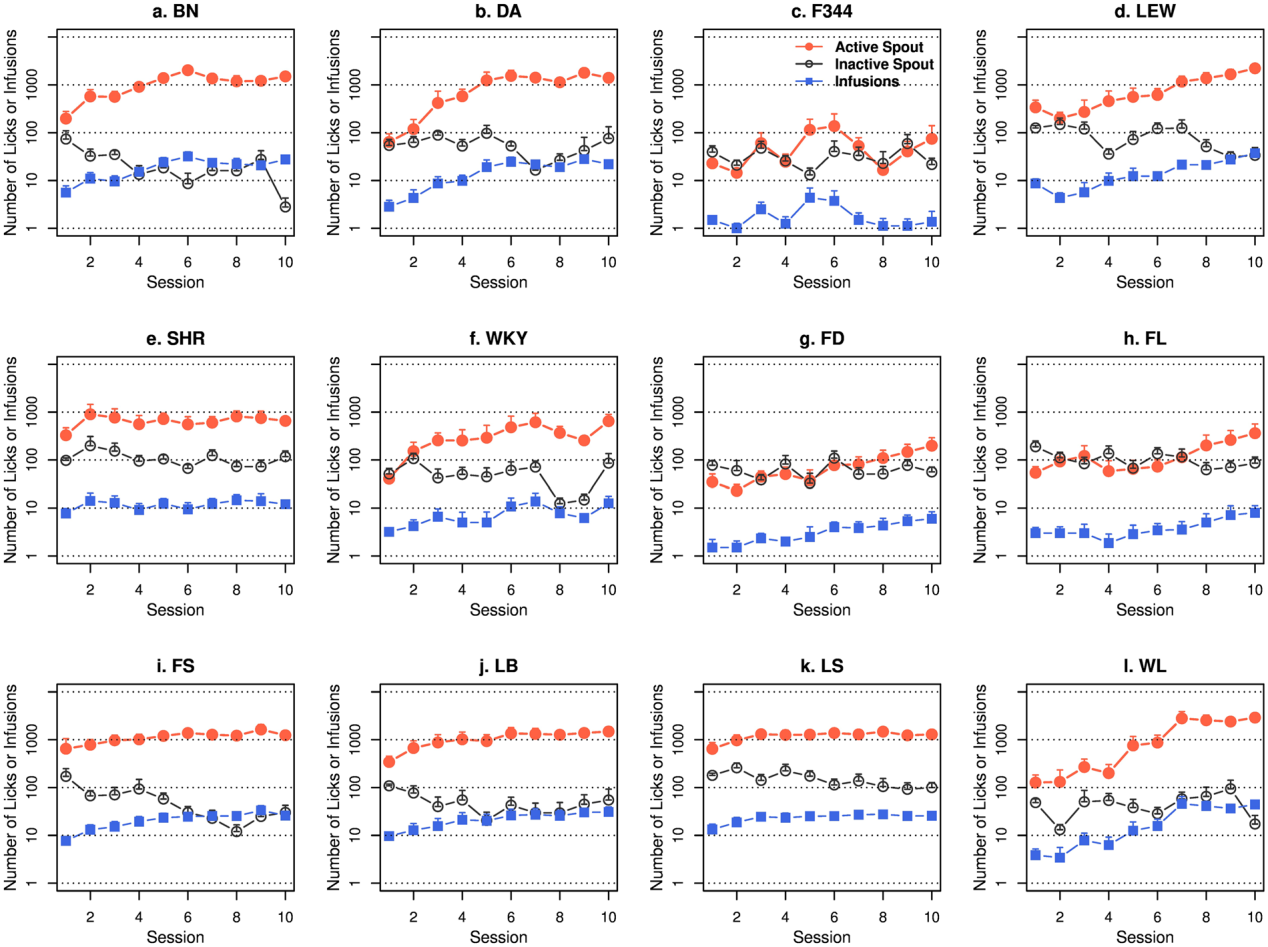
Female adolescent rats from 12 isogenic strains were tested by using the socially acquired nicotine (15 μg/kg/inf) IVSA procedure. See Methods for a detailed description of the procedure. A logarithmic scale is used for the Y-axis.

**Figure 2:**
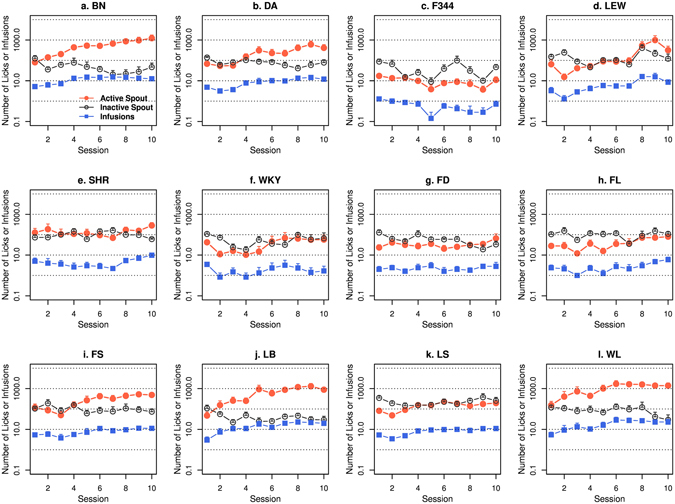
Female adolescent rats from 12 isogenic strains were tested by using the socially acquired nicotine (30 μg/kg/inf) IVSA procedure. See Methods for a detailed description of the procedure. The Y-axis used a logarithmic scale.

The numbers of nicotine infusions, licks on the active and inactive spouts during the ten nicotine IVSA sessions for the 12 isogenic strains are shown in Fig. 1 (15 μg/kg/inf) and Fig. 2 (30 μg/kg/inf). When 15 μg/kg/inf nicotine was provided, three strains (F344, FD, and FL) did not show a significant difference in the number of licks between the two spouts. All other strains licked more on the active spouts than on the inactive spouts (Table 1). At 30 μg/kg/inf nicotine, five strains licked more (BN, DA, FS, LB, and WL), and three strains (F344, FL, and LS) licked less on the active spout compared to the inactive spout. The other four strains did not show a main effect by spout (LEW, SHR, WKY, and FD) (Table 1).

**Table 1:** Statistical analysis of self-administration data: I. Spout difference.

Strain	Nicotine 15 μg/kg/inf.				Nicotine 30 μg/kg/inf.			
	n	Df	F	p	n	Df	F	p
BN	5	(1,40)	136.0	<**0.001**	5	(1,40)	47.5	<**0.001**
DA	6	(1,50)	75.1	<**0.001**	6	(1,50)	14.9	<**0.001**
F344	8	(1,70)	2.0	>0.05	7	(1,60)	15.5	<**0.001**
LEW	6	(1,50)	66.5	<**0.001**	7	(1,60)	1.4	>0.05
SHR	7	(1,60)	38.5	<**0.001**	5	(1,40)	2.0	>0.05
WKY	5	(1,40)	19.7	<**0.001**	6	(1,50)	1.9	>0.05
FD	6	(1,50)	1.5	>0.05	6	(1,50)	1.0	>0.05
FL	7	(1,60)	1.2	>0.05	8	(1,70)	15.8	<**0.001**
FS	7	(1,60)	135.9	<**0.001**	5	(1,40)	26.0	<**0.001**
LB	6	(1,50)	82.6	<**0.001**	5	(1,40)	46.9	<**0.001**
LS	5	(1,60)	148.2	<**0.001**	9	(1,80)	8.0	<**0.01**
WL	5	(1,60)	59.1	<**0.001**	7	(1,60)	46.6	<**0.001**

Twelve isogenic strains of rats self-administered nicotine (15 or 30 μg/kg/inf, i.v.) for 10 daily 3 h sessions with a flavored cue. A same-sex conspecific consuming the same cue served as a demonstrator to provide an environment for social learning. The number of active vs inactive licks were analyzed using two-way repeated measures ANOVA. The p values that achieved statistical significance (p < 0.05) are highlighted in bold.

Statistical analysis for the effect of the session on the number of licks and infusions is provided in Table 2 (15 μg/kg/inf) and Table 3 (30 μg/kg/inf). At 15 μg/kg/inf, eight strains (BN, DA, LEW, FD, FS, LB, LS, and WL) significantly increased the number of licks on the active spout across the sessions. Except FS, these strains also significantly increased the number of nicotine infusions across the sessions. At 30 μg/kg/inf, eight strains (except SHR, WKY, FD, and FL) increased the number of active licks across the sessions. Most of these strains (except FS) also significantly increased the number of nicotine infusions. F344 decreased the number of active licks and infusions across the sessions.

**Table 2:** Statistical analysis of self-administration data: IIa. Session effect, 15 μg/kg/inf nicotine.

Strains	n	Df	Infusion		Active spout		Inactive spout	
			F	p	F	p	F	p
BN	5	(9,36)	5.3	<**0.001**	5.3	<**0.001**	2.4	<**0.05**
DA	6	(9,45)	6.8	<**0.001**	5.9	<**0.001**	0.9	>0.05
F344	8	(9,63)	0.9	>0.05	0.7	>0.05	0.7	>0.05
LEW	6	(9,45)	12.4	<**0.001**	10.3	<**0.001**	1.7	>0.05
SHR	7	(9,54)	0.7	> 0.05	0.6	>0.05	0.8	>0.05
WKY	5	(9,36)	2.0	>0.05	1.8	>0.05	1.2	>0.05
FD	6	(9,45)	2.1	<**0.05**	2.4	<**0.05**	0.7	>0.05
FL	7	(9,54)	1.6	>0.05	1.9	>0.05	1.2	>0.05
FS	7	(9,54)	3.8	<**0.001**	1.7	>0.05	2.7	<**0.05**
LB	6	(9,45)	3.1	<**0.01**	2.7	<**0.05**	1.2	>0.05
LS	5	(9,54)	2.1	<**0.05**	2.1	<**0.05**	1.4	<**0.05**
WL	5	(9,54)	5.8	<**0.001**	6.3	<**0.001**	1.0	>0.05

Two-way repeated measures ANOVA was used to analyze the effect of session of the number of nicotine infusions and licks on the active or the inactive spout.

**Table 3:** Statistical analysis of self-administration data: IIb. Session effect, 30 μg/kg/inf nicotine.

Strains	n	Df	Infusion		Active spout		Inactive spout	
			F	p	F	p	F	p
BN	5	(9,36)	5.2	<**0.001**	3.2	<**0.01**	1.4	>0.05
DA	6	(9,45)	4.0	<**0.001**	2.8	<**0.01**	1.5	>0.05
F344	8	(9,54)	2.2	<**0.05**	2.1	<**0.05**	1.4	>0.05
LEW	6	(9,54)	3.7	<**0.01**	2.1	<**0.05**	2.4	<**0.05**
SHR	7	(9,36)	1.8	>0.05	0.7	>0.05	1.3	>0.05
WKY	5	(9,45)	1.3	>0.05	1.1	>0.05	1.2	>0.05
FD	6	(9,45)	1.1	>0.05	1.1	>0.05	2.1	>0.05
FL	7	(9,63)	2.5	<**0.05**	1.8	>0.05	0.9	>0.05
FS	7	(9,36)	1.7	>0.05	3.1	**<0.01**	0.7	>0.05
LB	6	(9,36)	3.6	**<0.01**	3.4	**<0.01**	2.2	**<0.05**
LS	5	(9,72)	3.3	**<0.01**	2.2	**<0.05**	1.0	>0.05
WL	5	(9,54)	5.1	**<0.001**	4.9	**<0.001**	0.8	>0.05

Two-way reapeated measures ANOVA was used to analyze the effect of the session of the number of nicotine infusions and licks on the active or the inactive spout.

**Figure 3:**
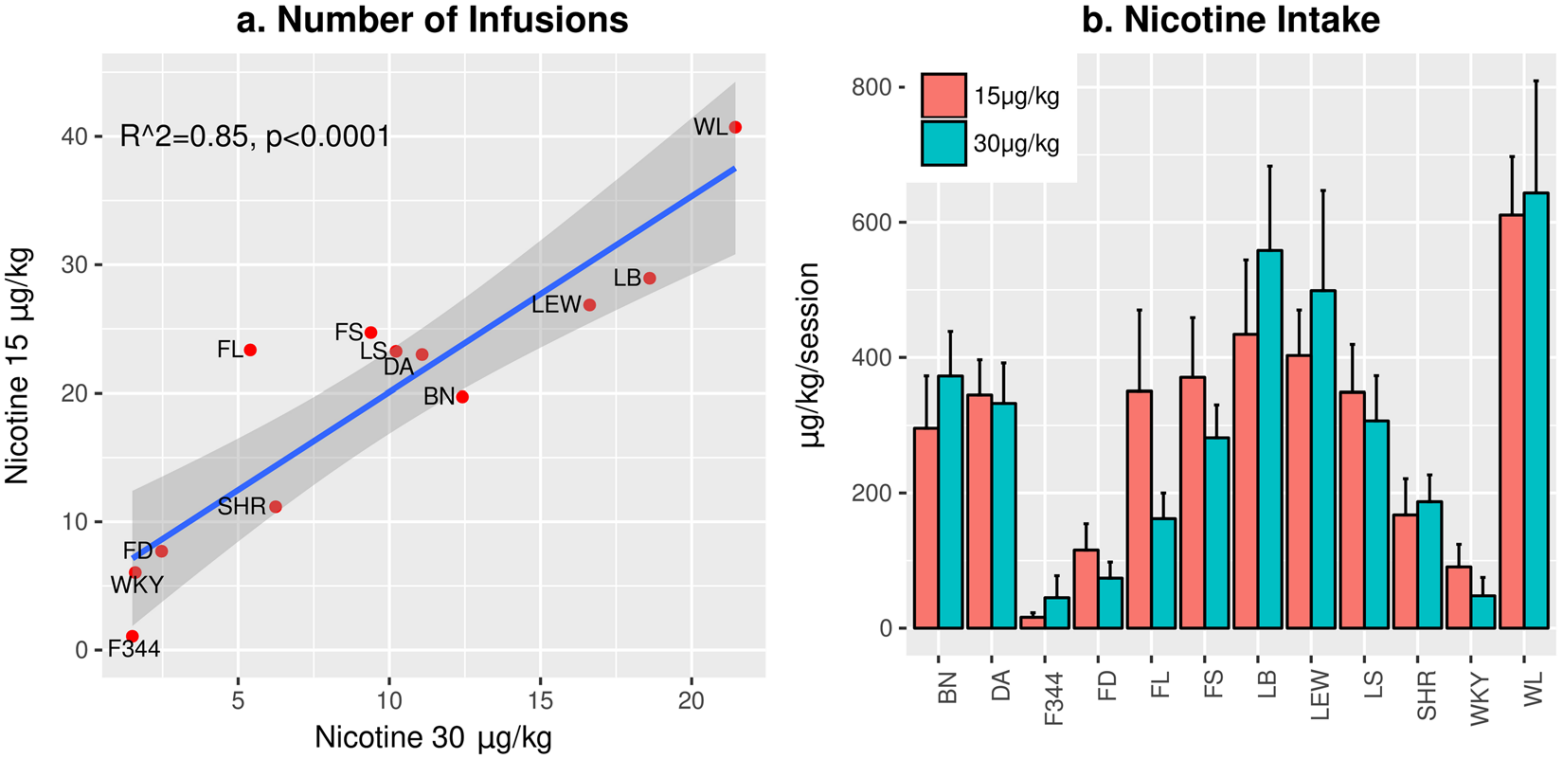
Average nicotine intake during the last three IVSA sessions. The number of nicotine infusions was highly correlated when two different doses were tested (**a**). The gray area represents the 95% confidence interval of the linear model. When compared to the 30 μg/kg/inf dose, the number of infusions almost doubled across the strains when 15 μg/kg/inf was used. As a result, the amount of nicotine obtained was very similar between the two doses (**b**). Three-way repeated measures ANOVA found there was a significant effect of strain on nicotine intake (F_11,160_ = 9.0, p < 0.001) but dose (F_1,166_ = 0.13, p > 0.05) and session (F_2,320_ = 0.17, p > 0.05) had no effect.

We found a strong correlation between the average number of nicotine infusions obtained when the two nicotine doses were tested across the 12 isogenic strains (Pearson coefficient = 0.917, p < 0.0001. Figure 3a). Further, the amount of nicotine intake was very similar between the two doses across the strains (Fig. 3b. F_1,159_ = 1.06, p > 0.05 for the effect of dose). On average, the ratio of nicotine intake at 30 μg/kg/inf. was 1.05 ± 0.1-fold of that at 15 μg/kg/inf. The stable nicotine intake at difference doses across 12 strains strongly suggested that nicotine, rather than the flavor cue, is the primary reinforcer of the operant behaviors.

**Figure 4:**
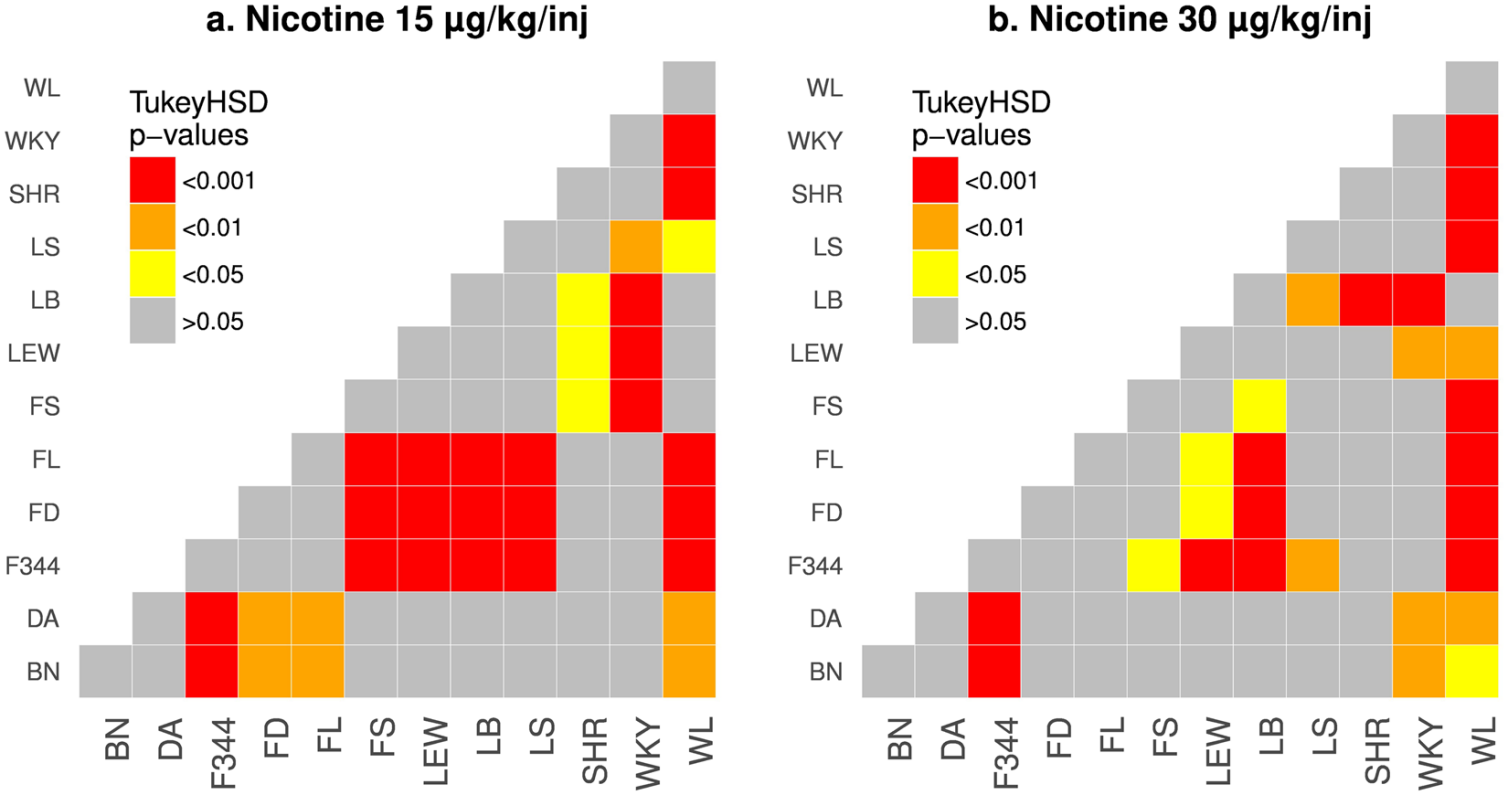
Strain difference. Strain differences in the average number of nicotine infusions obtained during the last three IVSA sessions were analyzed by post-hoc Tukey HSD tests. Each color block represented the p-value when the two strains listed on the x and y-axis were compared.

We further compared the amount of nicotine intake during the last three IVSA sessions between the strains. Post-hoc analysis using Tukey HSD showed numerous significant strain differences. These results are provided in Fig. 4. The strain differences are very similar between the 15 and 30 μg/kg/inf doses, although the lower does tends to be more sensitive in detecting strain differences. Overall, these data suggested that genetic factors had a large effect on nicotine intake. We calculated the narrow sense heritability (h^2^) for nicotine intake to be 0.54 for 15 μg/kg/inf and 0.65 for 30 μg/kg/inf.

### Conditioned flavor aversion

**Figure 5:**
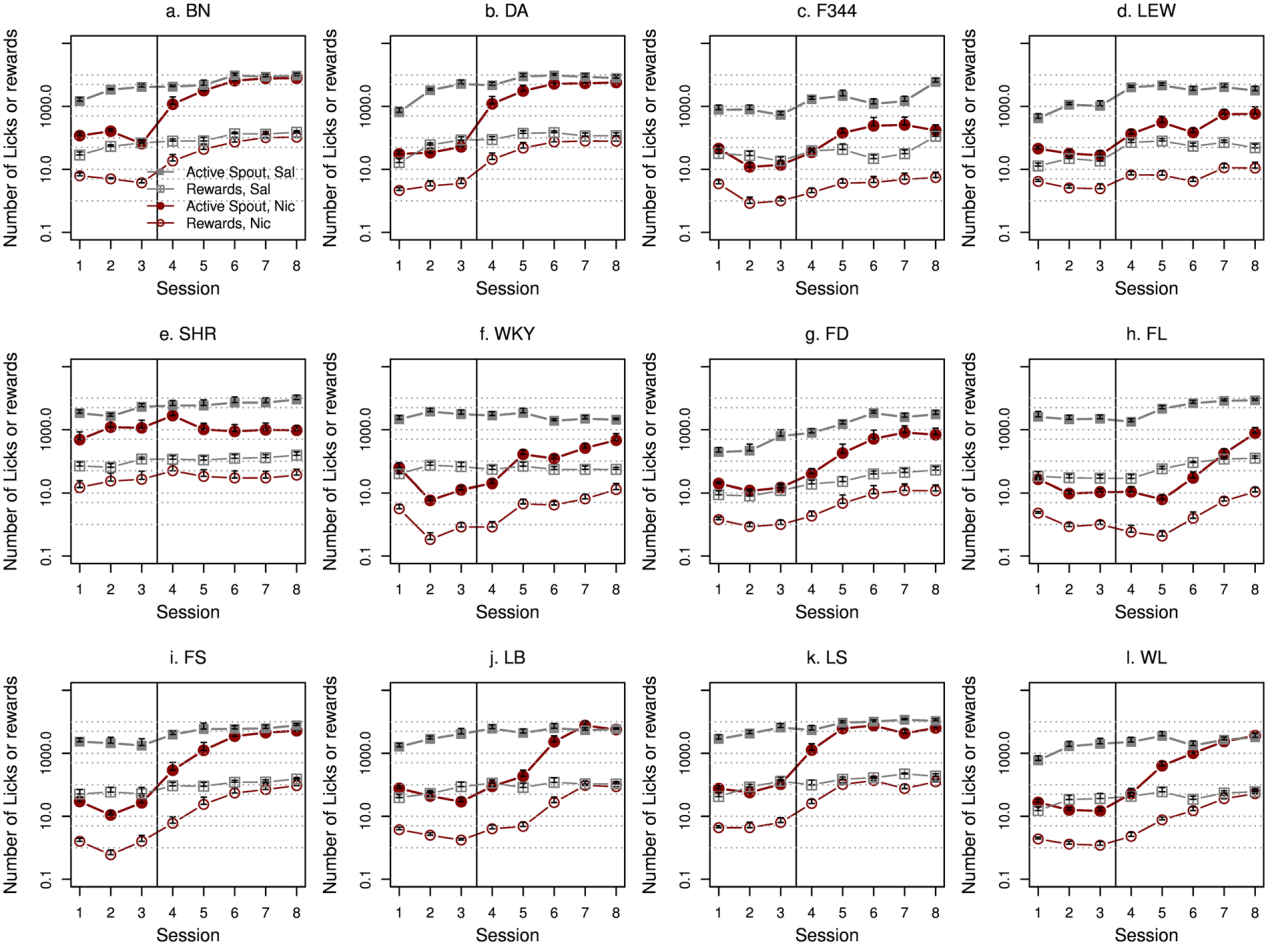
Nicotine CFA and extinction of CFA. Twelve isogenic strains of adolescent rats self-ministered nicotine (30 μg/kg/inf, i.v.) with an appetitive flavor cue for three daily sessions without demonstrator rats (sessions 1–3). This was followed by five daily extinction sessions (sessions 4–8) where nicotine was not provided. Control rats received i.v. saline.

Our previous study showed that rats developed conditioned aversion to the flavor associated with self-administered nicotine^8^. We tested CFA after three nicotine (30 μg/kg/inf) IVSA sessions. Control rats received i.v. saline (Fig. 5). The data collected during the first three IVSA sessions showed that all 12 isogenic strains emitted significantly fewer licks on the active spout and obtained fewer infusions when nicotine was provided, compared to the saline groups (Table 4), indicating that nicotine was aversive. Ten strains (except SHR, and LS) showed significant CFA when tested on the fourth session (Table 4). After four more extinction sessions (i.e., rats received the flavor cue but not nicotine), six strains (BN, DA, FS, LB, LS, and WL) extinguished the CFA response (i.e., the number of active licks and saccharin reward were not statistically different between the saline and nicotine groups in these strains. Table 4).

**Table 4:** Effect of nicotine on the acquisition of socially acquired nicotine IVSA, CFA, and extinction of CFA.

Strain	n1, n2	Acquisition of IVSA						CFA test		Extinction of CFA	
		Active Licks			Infusions			Active Licks	Rewards	Active Licks	Rewards
		Df	F	p	Df	F	p	p	p	p	p
BN	(4,3)	(1,5)	309.5	**<0.001**	(1,5)	158.7	**<0.001**	**<0.05**	**<0.05**	>0.05	>0.05
DA	(7,4)	(1,9)	60.4	**<0.001**	(1,9)	127.3	**<0.001**	**<0.05**	**<0.05**	>0.05	>0.05
F344	(6,4)	(1,8)	58.7	**<0.001**	(1,8)	13.5	**<0.01**	**<0.01**	**<0.05**	**<0.01**	**<0.05**
LEW	(7,5)	(1,10)	160.0	**<0.001**	(1,10)	26.9	**<0.001**	**<0.001**	**<0.05**	**<0.01**	>0.05
SHR	(7,4)	(1,9)	8.1	**<0.05**	(1,7)	7.8	**<0.05**	>0.05	>0.05	**<0.05**	**<0.05**
WKY	(6,5)	(1,9)	211.6	**<0.001**	(1,9)	14.8	**<0.01**	**<0.001**	**<0.05**	**<0.05**	**<0.01**
FD	(7,5)	(1,10)	12.3	**<0.01**	(1,10)	9.5	**<0.05**	**<0.01**	**<0.05**	**<0.05**	**<0.05**
FL	(7,5)	(1,10)	128.8	**<0.001**	(1,10)	14.5	**<0.01**	**<0.001**	**<0.05**	**<0.01**	**<0.01**
FS	(5,4)	(1,7)	113.9	**<0.001**	(1,7)	8.5	**<0.05**	**<0.05**	**<0.05**	>0.05	>0.05
LB	(4,4)	(1,6)	63.2	**<0.001**	(1,6)	13.4	**<0.05**	**<0.001**	**<0.05**	>0.05	>0.05
LS	(6,5)	(1,9)	44.3	**<0.001**	(1,9)	14.0	**<0.01**	>0.05	>0.05	>0.05	>0.05
WL	(6,6)	(1,10)	110.1	**<0.001**	(1,10)	17.4	**<0.01**	**<0.01**	**<0.05**	>0.05	>0.05

Two groups of rats received i.v saline or nicotine with contingent flavor cue in the abscence of demonstrator rats for each strain. n1 and n2 are the number of animals used for the nicotine and saline groups, respectively. The number of licks on the active spout and infusions were compared between the two treatment groups (i.e., nicotine vs saline) for each strain using two-way repeated measures ANOVA. These rats then were tested for CFA during the fourth session (i.e. CFA test). CFA tests continued for a total of five daily sessions and the data on the last session were used to evaluate the extinction of CFA. Two-tailed independ t-tests were used to compare the effect of treatment on CFA and extinction of CFA.

### Correlation between CFA, extinction of CFA, and nicotine intake

**Figure 6:**
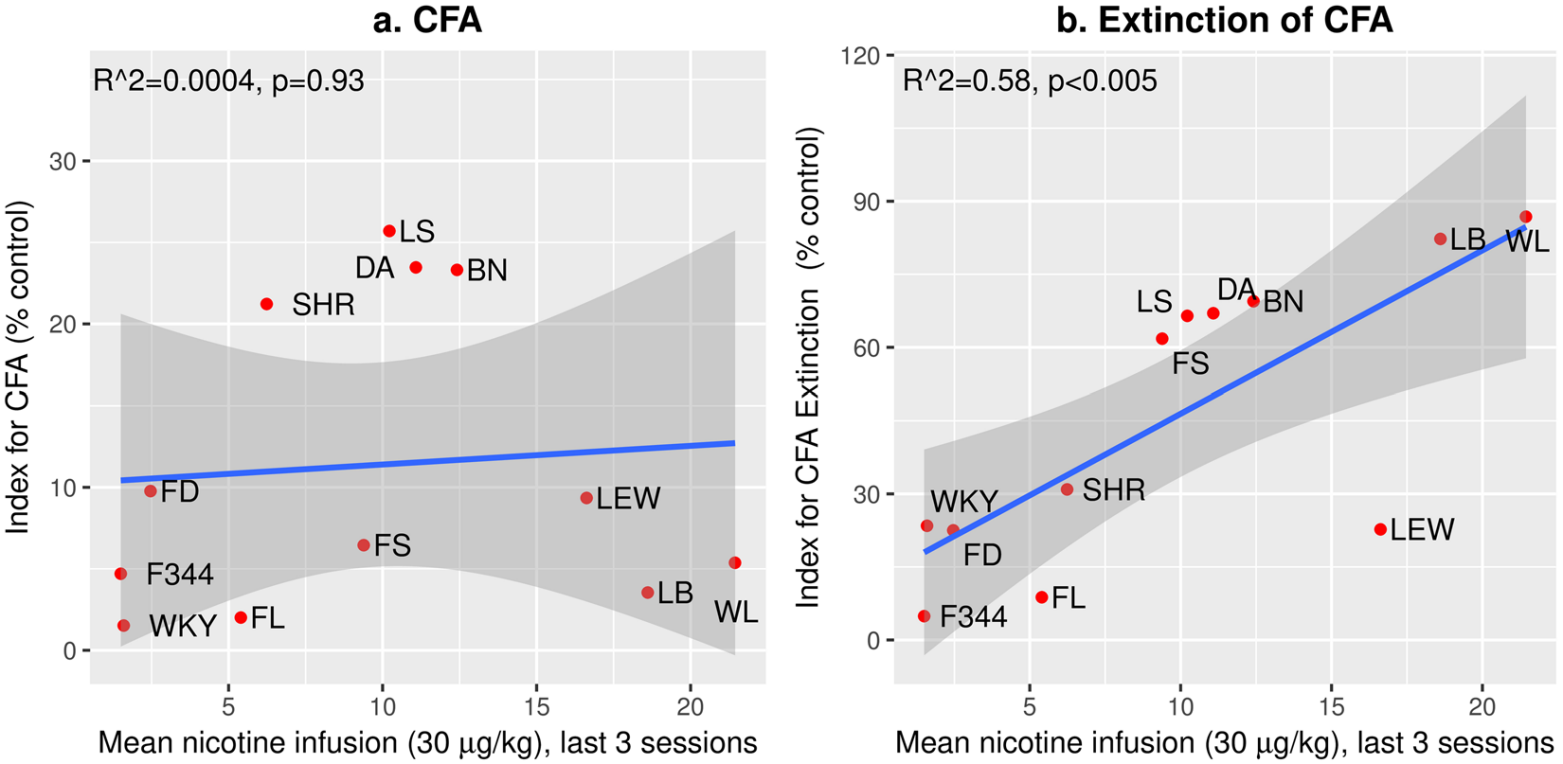
Correlation between nicotine intake and indices for CFA and extinction of CFA. The CFA index (**a**) was calculated using data obtained from the first extinction session (i.e. session four, Fig. 5). The extinction index (**b**) was calculated using data obtained from session eight. The correlation between the number of nicotine infusions at 30 μg/kg/inf with both indices were calculated. The gray area represents the 95% confidence interval of the linear model.

We correlated the amount of nicotine intake and a CFA index across the 12 strains (Fig. 6a). Pearson correlation coefficient was -0.02 (R^2^ = 0.004, p = 0.93), indicating that the amount of nicotine intake was not correlated with the degree of CFA. However, there was a significant correlation between nicotine intake and the index for CFA extinction. The Pearson coefficient was 0.76 (R^2^ = 0.58, p < 0.005, Fig. 6b). This strong correlation indicated that the extinction of CFA is a main factor controlling nicotine intake. These data suggest that facilitating the extinction of nicotine CFA is a likely mechanism by which social learning promotes nicotine intake.

## Discussion

We tested the socially- acquired nicotine IVSA model using 12 isogenic strains of adolescent rats. Our data showed that there was a large variation in the level of nicotine intake among these strains, ranging from an average of 1.8 to 42.4 infusions per 3 h session during the last three sessions. The amount of self-administered nicotine was very similar for each strain when two different doses of nicotine were provided, indicating strong self-regulation in drug intake. We estimated the heritability of nicotine intake to be 0.54–0.65. Although we showed that nicotine induced CFA in most of these strains, nicotine intake was not correlated with the degree of CFA. Instead, nicotine intake was correlated with the extinction of CFA, which suggested that the role of social learning is likely in facilitating the extinction of conditioned aversive response to nicotine.

We previously demonstrated many key features of the socially acquired nicotine self-administration model. For example, we have shown that strong aversive effect of nicotine can be overcome by social learning^8^. We also showed that the odor, rather than the taste cue associated with nicotine, is critical for social learning^8^. In addition, a gaseous component of the rodent breath, carbon disulfide, is needed for the effect of social learning^9^. We have further shown that social learning can facilitate nicotine IVSA even when the flavor cue is aversive (bitter)^17^, indicating that 1) nicotine has a strong reinforcing effect that is independent on the subjective value of the flavor cue and 2) social learning is a powerful modulator of reward. In addition to confirming many of these findings, the new data presented here demonstrated two additional salient aspects of our model: 1) the effect of social learning is likely in facilitating the extinction of nicotine CFA and 2) genetic factors play a critical role in nicotine intake in this model.

The involvement of social factors in the initiation of smoking has been well documented. For example, the majority of first-time cigarette uses usually occur in the presence of friends, and peer smoking is one of the strongest predictors of smoking initiation^18, 19^. However, mechanistic understanding of the role of social environment on smoking behavior is still lacking. By demonstrating the role of social learning in nicotine intake in rodents, our model provides a great opportunity to explore the mechanisms underlying the interaction between social factors and cigarette smoking.

Numerous human genetics studies have identified that variations in the CHRNA3-CHRNA5-CHRNAB4 cluster on chromosome 15 are associated with several aspects of the smoking behavior^20, 21^. Animal studies have shown that chrna5 is associated with the aversive effect of nicotine. Thus, we anticipated that the amount of nicotine intake in our model correlated with the aversive response measured by CFA. To our surprise, our data showed no such correlation (Fig. 6a). One likely reason is that our procedure models the acquisition phase of smoking, not chronic smoking, which is investigated in human genetic studies. Thus, although most strains developed conditioned aversive response to nicotine, it is not a strong determinant of nicotine intake during the early stages of nicotine consumption.

Several reports have shown that a social environment per se can be rewarding for rats in general^22, 23^. However, the data shown in Fig. 5b indicated that a potential function of the social environment has a more specific role in our model, which is to facilitate the extinction of conditioned aversive response to nicotine. This idea is in agreement with our previous finding that a social environment *per se* is not sufficient to promote nicotine intake with a flavor cue^8^. The distinction between enhancing reward vs. enhancing extinction of CFA is critical, because brain regions underlying these processes are likely to differ. For example, the mesolimbic circuit is likely involved in enhancing reward. On the other hand, many studies have shown that the infralimbic cortex is involved in the extinction of conditioned aversive stimuli, such as fear^24^. The infralimbic cortex also has been implicated in the extinction of cocaine^25^ and heroine-seeking behavior^26^. Therefore, our data suggested that the infralimbic cortex could potentially promote drug intake by facilitating the extinction of conditioned aversive response to the drug. We are currently investigating this hypothesis.

One limitation of this study is that we included only female rats. Possible sex differences in nicotine IVSA is a critical aspect of the animal models^27^ and have been studied by many research groups. For example, Lynch^28^ showed more females acquired nicotine IVSA than males. Swalve *et al*.^29^ found the opposite trend in acqusition but no sex difference in nicotine infusion post-acquisition. Chaudhri *et al*.^30^ studied the sex difference of nicotine IVSA using three nicotine doses (0.03, 0.06, 0.15 mg/kg) and found females obtained more nicotine when 0.06 mg/kg was used. However, Feltenstein^31^ showed there was no sex difference in nicotine IVSA when 0.03 or 0.05 mg/kg was used, and no estrous cycle dependent changes. Donny *et al*.^32^ showed that nicotine intake was similar between males and females but the motivation to obtain nicotine was higher in females (no effect of estrous cycle). More recently, Peartree *et al*.^33^ showed that a social context enhances the initial reinforcing effects of nicotine in males, but protects against nicotine intake during later sessions especially in females (again, estrous cycle had no effect). We have shown that in adolescent Sprague-Dawley rats, there was no sex difference in nicotine IVSA using either a 23-h access paradigm (lever pressing)^34^ or the socially acquired model^8^. However, in heterogenous stock rats, females self-administered significantly more nicotine than did males when the social model was used^35^. Thus, sex differences in nicotine IVSA are likely sensitive to the experimental condition and genetic background.

We have found many strain differences in nicotine intake in this study (Fig. 4). Most strains used in this study were also investigated in a previous report where nicotine self-administration was conducted using a lever response^16^. Although the estimated heritability of nicotine intake is very similar between these two studies, nicotine intake was very different for most strains. One potential difference is sex difference because only females were used in this study as discussed above. However, a more likely cause is the different cues used for nicotine intake: while a flavor cue and a social learning environment were provided in this study, a visual cue was used in our previous study, and rats were tested in a non-social setting. A third potential difference is that rats used in our previous experiment were food deprived and trained to press the lever for a food reward before nicotine was made available. In contrast, rats were not deprived of food or water in the socially acquired nicotine IVSA model, and thus were less likely to be affected by the motivational effect introduced by food restriction^36^.

Despite these differences, the F344 strain consistently had the lowest nicotine intake, while the LEW strain took moderate to high amounts of nicotine in both studies. This is consistent with a few studies that compared the effect of nicotine between these two strains. For example, F344 did not acquire nicotine IVSA in a 23-h access model, while the LEW strain did^37–39^. This difference is likely specific to nicotine, because these strains had similar behavior profiles when food reward was provided^38^; they also have a similar profile when tested for conditioned taste aversion included by LiCl^40^. Interestingly, the LEW strain is more sensitive to mecamylamine-precipitated withdrawal aversion than was the F344 strain^41^. Thus, one potential mechanism that can potentially reconcile these data is the hypothesis that the F344 strain has a strong acute aversive response to nicotine, as shown in Fig. 5, day 4. This effect is likely a dominant genetic effect, because the F1 crosses FD, FS, and FL all showed strong CFA, while minimal CFA was shown for the DA and SHR strains.

Together, these data showed that nicotine intake in the socially acquired nicotine IVSA model is under the control of the reward as well as the aversive effects of nicotine. The rewarding effect of nicotine was shown by the increased operant response when a lower dose (15 μg/kg/inf) of nicotine was given. The effect of the aversive property was shown by the strong CFA in most strains. In addition, social learning facilitates the extinction of CFA. By dissociating the aversive effect of nicotine and its cue (i.e., the oral flavor), social learning steers the balance towards a greater amount of nicotine intake. Last, the large strain difference indicated that there is a strong genetic control of nicotine intake.

## Methods

### Animals

Breeders of six inbred strains of rats, including Brown Norway (BN), Dark Agouti (DA), Fisher 344 (F344), Lewis (LEW), Spontaneously Hypertensive Rat (SHR), and Wistar Kyoto (WKY) were obtained from Harlan Laboratories (Indianapolis, IN). Rats were housed in a 12:12 h reversed light cycle (lights off at 9:00 am) with food and water available *ad libitum*. All animals for the IVSA experiments were bred on site. Inbreeding was maintained within each strain. As shown in Table 1, six selected F1 hybrids were also generated: LEW-BN (LB), F344-LEW(FL), F344-SHR (FS), WKY-LEW (WL), F344-DA (FD), LEW-SHR (LS). These F1s were selected based on the nicotine intake of the inbred strains reported previous^16^. The two letters for each F1 hybrid represented the initials of the maternal and paternal strains, respectively. Only adolescent rats were used because most smokers start smoking during adolescence and because our previous study showed that adolescent rats acquired nicotine IVSA at a faster rate and maintained higher nicotine intake than did adult rats^34^. Only female offspring were used because males were used in another study^16^. All procedures were approved by the Institutional Animal Care and Use Committee of the University of Tennessee Health Science Center. All procedures followed the NIH Guide for the Care and Use of Laboratory Animals.

### Nicotine IVSA

Procedure for the socially acquired nicotine IVSA has been described in detail^8^. The surgery for inserting a jugular catheter and a 3D printed implant was described previously^42^. Surgery was conducted on postnatal day 38–40 under isoflurane anaesthesia. Ketoprofen (2 mg/kg, s.c.) was given for postoperative analgesia. The jugular catheter was flushed daily using 0.1 ml heparinized saline (100 IU/ml) and 0.1 ml Baytril (3 mg/kg) for 3 days after the surgery. Rats were then given access to nicotine IVSA sessions 3 h per day for 10 days in the dark-phase of the light cycle.

A detailed illustration of the operant chamber (Med-Associates, Inc.) has been provided elsewhere^35^. Each self-administration rat was paired with a randomly chosen conspecific (i.e., a demonstrator rat) during the experiment. These two rats were separated by a transparent plastic panel with six round holes that allow orofacial interactions. Two drinking spouts were installed on the side of the self-administration rat. Two syringe pumps were placed outside the sound attenuating chamber; one delivered nicotine (i.v.) through a swivel located on top of the chamber, and the other delivered the flavor cue (0.4% saccharin and 0.1% unsweetened grape-flavored Kool-Aid) to the active spout. The inactive spout contained no solution. Only one spout was available on the side of the demonstrator rat. A bottle installed on top of the spout provided unrestricted access to the same flavor solution to the demonstrator rat. The demonstrator rat received no nicotine injection. Each spout was connected to a contact lickometer controller allowing the number and the timing of licks to be recorded. IVSA was conducted using a fixed-ratio 10 schedule with 20 s timeout period. Thus, ten licks on the active spout activated the delivery of a 60 μl flavor cue, and an i.v. infusion (nicotine, free base, 15 or 30 μg/kg/inf., or saline).

### Conditioned flavor aversion (CFA)

Two groups of rats were used for each strain. One group received nicotine (30 μg/kg/inf, i.v.), the other group received i.v. saline. Each group received three daily IVSA sessions as described above, with the exception that no demonstrator rats were provided. This was followed by five CFA test sessions where licking resulted in the delivery of the flavor cue but no i.v. infusion was given. We calculated the CFA index as the ratio of the reward earned (i.e., drops of the flavor cue) of the nicotine group to that of the saline controls in the first CFA test (i.e., session four). In addition, we also calculated the extinction index of CFA, which used the same calculation method but the data were obtained from the last CFA test session.

### Statistical analysis

The number of licks was transformed to a log scale so that the data fit a normal distribution. Data are presentes as Mean ± SEM. Two-way repeated measures ANOVA was used to analyze the effect of strain on the number of nicotine infusions and licks, as well as the effect of the spout on the number of licks. Both session (i.e., day) and spout were treated as within-subject variables. The correlation between nicotine infusion during the last three IVSA sessions, the CFA index and the extinction index were calculated. All inbred and F1 crosses are isogenic, permitting the calculation of the narrow sense heritability. The between-strain variance provided a measure of additive genetic variation (VA), while within-strain variance represented environment variability (VE). An estimate of narrow-sense heritability (i.e., the proportion of total phenotypic variation that is due to the additive effects of genes, h^2^) was obtained using the formula: h^2^ = VA/(VA + VE). Statistical analyses were performed using the R statistical language. Statistical significance was assigned when p < 0.05.
